# Biosynthesis of silver nanoparticles for the fabrication of non cytotoxic and antibacterial metallic polymer based nanocomposite system

**DOI:** 10.1038/s41598-021-90016-w

**Published:** 2021-05-18

**Authors:** Sadaf Raza, Asma Ansari, Nadir Naveed Siddiqui, Fariha Ibrahim, Muhammad Ishaque Abro, Afsheen Aman

**Affiliations:** 1 The Karachi Institute of Biotechnology and Genetic Engineering (KIBGE), University of Karachi, Karachi, 75270 Pakistan; 2 Department of Metallurgy and Material Engineering, Mehran University of Engineering and Technology (MUET), Jamshoro, Pakistan

**Keywords:** Biomaterials, Nanobiotechnology

## Abstract

Nanomaterials have significantly contributed in the field of nanomedicine as this subject matter has combined the usefulness of natural macromolecules with organic and inorganic nanomaterials. In this respect, various types of nanocomposites are increasingly being explored in order to discover an effective approach in controlling high morbidity and mortality rate that had triggered by the evolution and emergence of multidrug resistant microorganisms. Current research is focused towards the production of biogenic silver nanoparticles for the fabrication of antimicrobial metallic-polymer-based non-cytotoxic nanocomposite system. An ecofriendly approach was adapted for the production of silver nanoparticles using fungal biomass (*Aspergillus fumigatus* KIBGE-IB33). The biologically synthesized nanoparticles were further layered with a biodegradable macromolecule (chitosan) to improve and augment the properties of the developed nanocomposite system. Both nanostructures were characterized using different spectrographic analyses including UV–visible and scanning electron microscopy, energy dispersive X-ray analysis, dynamic light scattering, and Fourier transform infrared spectroscopic technique. The biologically mediated approach adapted in this study resulted in the formation of highly dispersed silver nanoparticles that exhibited an average nano size and zeta potential value of 05 nm (77.0%) and − 22.1 mV, respectively with a polydispersity index of 0.4. Correspondingly, fabricated silver–chitosan nanocomposites revealed a size of 941 nm with a zeta potential and polydispersity index of + 63.2 mV and 0.57, respectively. The successful capping of chitosan on silver nanoparticles prevented the agglomeration of nanomaterial and also facilitated the stabilization of the nano system. Both nanoscopic entities exhibited antimicrobial potential against some pathogenic bacterial species but did not displayed any antifungal activity. The lowest minimal inhibitory concentration of nanocomposite system (1.56 µg ml^−1^) was noticed against *Enterococcus faecalis* ATCC 29212. Fractional inhibitory concentration index of the developed nanocomposite system confirmed its improved synergistic behavior against various bacterial species with no cytotoxic effect on NIH/3T3 cell lines. Both nanostructures, developed in the present study, could be utilized in the form of nanomedicines or nanocarrier system after some quantifiable trials as both of them are nonhazardous and have substantial antibacterial properties.

## Introduction

Nanocomposite is a unique class of multiphase nanostructured entities that should have at least one phase in nanoscale dimension. They exhibit unique physiochemical properties that makes them a plausible competitor against various synthetic therapeutic agents hence, they could be exploited as a nanocarrier for drug delivery system. There are numerous types of nanocomposites that are generally classified on the basis of their unique structure and matrix type. Some of the nanocomposites are recognized as polymer-based nanocomposites while, others are categorized as non-polymer-based nanocomposites^1–3^. Among various types of polymer-based nanocomposites, metallic nanocomposites (metal/polymer nanocomposites) have gained massive attention due to their enormous range of applications. The polymer-based nanocomposites are generally a combination of either organic polymers or a specific biomacromolecule in which an inorganic nanofiller is flawlessly embedded. Some metallic nanocomposites are widely explored not only because they are versatile, environmentally friendly but are biodegradable, and biocompatible in nature^4,5^. These exceptional nanomaterials have unique physicochemical properties and are renewable in nature^6^. Particularly, in metallic based nanocomposite systems, a variety of metallic nanoparticles have been investigated as nano-filler substances because these nanosized metallic particles could get evenly dispersed within a polymer-based matrix. Among them, some of the metallic nanoparticles have also previously exhibited distinctive physical, chemical, and biological properties along with the extraordinary antimicrobial properties even in very low concentrations. Very frequently, silver, copper and gold nanoparticles have earlier been used as potent antimicrobial agents against a broad range of microorganisms^7–10^. Although, metallic nanoparticles can be synthesized using different chemical procedures with either bottom-up or top-down approach but in order to counteract any hazardous consequences of conventional procedures, biologically mediated biosynthesis platform could be adapted. In green biosynthesis, generally plant extracts, bacteria, fungi or algae are used^11^. Moreover, whatever the means of synthesis has been adapted, bare metallic nanoparticles sometimes do pose threat to the biotic systems when used alone. Therefore, to diminish any cytotoxic effect of metallic nanoparticles and additionally to improve their antimicrobial potential, they are layered with a biocompatible organic matrix. This particular strategy has led towards the development of a variety of metallic nanocomposites systems. Among naturally occurring biopolymers, chitosan is an exopolysaccharide that belongs to the family of aminopolysacchride and have exhibited credible antimicrobial potential along with numerous biomedical applications. Owing to the multiple functionalities, both the metallic nanoparticles and the biopolymers have successfully gained considerable attention of the investigators in order to use them in the field of biomedicine for efficient control of different infections that are caused by multidrug resistant microorganisms (MDROs).

The evolution of commensal bacteria into MDROs is a serious health concern and a major cause of high morbidity and mortality rate throughout the world. In this context, the majority of the available conventional antibiotics have lost their potential efficacy to treat microbial infections therefore, the exploration of alternative therapeutics to treat life-threatening diseases, which are mostly acquired through hospital infections, have become a global concern. Therefore in recent years, alternative approaches are being pursued in the form of nanomaterials as an important factor in order to eradicate issues associated with MDROs^16,17^. Recently, few studies have also highlighted the therapeutic applications and the usefulness of the metallic nanocomposites as an alternative approach against multidrug resistance^18,19^. Keeping the current scenario in view, the present study was hypothesized that the fabrication of an antimicrobial non-cytotoxic polymer-based nanocomposite system could help in the development of a nanocarrier drug delivery vehicle in order to control emerging cases of MDROs. In the emerging field of nanoscience and exclusively in nanobiotechnology, nanocomposites have recently gained considerable attention because of their unique multiphasic trait^4,20^ and also because there have been endless revolutionary developments in this particular area. This success was specifically achieved by modifying different types of nanomaterials and colloids.

The current research was designed initially to biosynthesize metallic nanoparticles using a fungal strain and use these biologically synthesized silver nanoparticles (AgNPs) for the development of silver–chitosan nanocomposite system (AgCNCs). In this study, an organic polymer known as chitosan was used as a coating matrix. Both the biosynthesized AgNPs and the fabricated AgCNCs were completely characterized in detail using different analytical techniques. To ascertain the harmonious effect and the potential application of the fabricated nanocomposite system, their antimicrobial potential and cytotoxicity levels were also investigated.

## Results and discussion

Functionalized nanomaterials are the key elements that could be used as an alternative therapeutic entities in the field of medical sciences. Every year, a large number of populations are exposed to MDROs, which are difficult to treat when conventional medicines or treatments are used. Previously, some studies have also elucidated the importance of nanotechnology controlling MDROs by conjugating different types of metallic nanoparticles with diverse polymers and pharmaceutical agents. Metallic nanoparticles have a huge potential to serve as a nanocarrier system during medical treatments for the controlled release of a specific drugs at the site of infection^21,22^. Large surface area and unique characteristic features also makes them a plausible candidate for the advanced research studies and equally in the treatment of microbial infections. However, their consistent use could result in the development of toxicity within a biological system and therefore, may cause hindrance in the actual treatment of MDROs related infections. Along with this, some of the nanoparticles are equally unstable when they are introduce in a metabolic system. Therefore, to minimize the risk of toxicity and to also escalate the stability of the metallic nanoparticles within a biological system, they could be coated or layered with a diverse range of naturally obtainable biocompatible polymers. The amalgamation of metallic nanoparticles with natural biopolymers has directed the development of metallic/biopolymer-based nanocomposite system. Some biopolymers are biodegradable, and their ubiquitous nature makes them a promising candidate for the fabrication of diverse range of nanocomposite systems, such as metallic, ceramic, or clay based nano-systems. Metallic biopolymer-based nanocomposite systems could be used as an alternative approach to regulate microbial infectious because they exhibit additional stability, low cytotoxicity and intensified combinatorial effect against MDROs.

**Figure 1:**
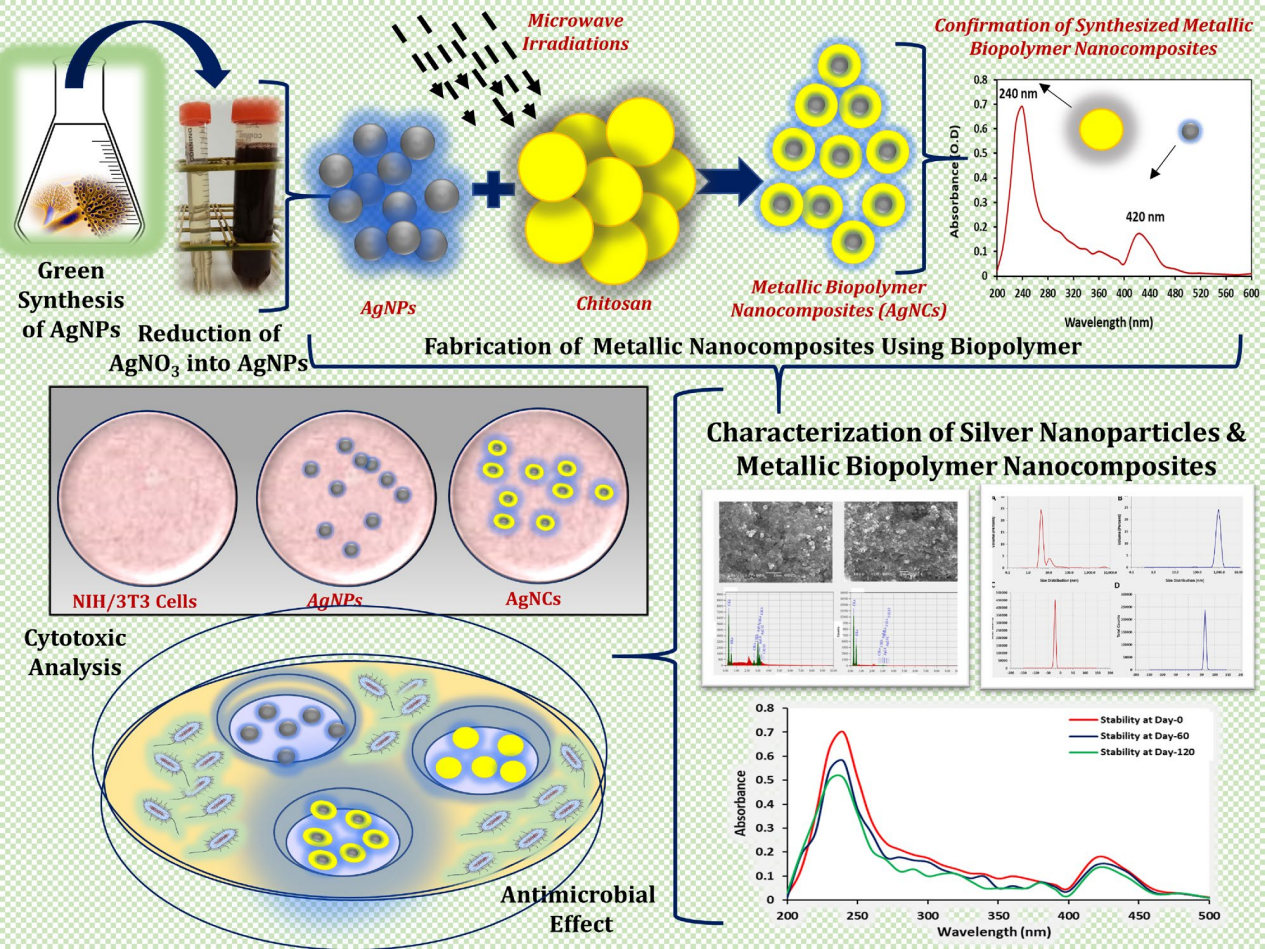
Schematic illustration of the biogenic synthesis and characterization of silver nanoparticles (AgNPs) and the fabricated nanocomposite system (AgCNCs).

Current study is an attempt to fabricate metallic-polymer-based nanocomposite system (silver nanoparticle–chitosan based nanocomposites or AgCNCs) that would have an antimicrobial potential against different pathogenic microorganisms with minimal or no cytotoxic impact. Figure 1 represents the overall schematic presentation of the designed study that was undertaken. To accomplish this goal, low molecular weight chitosan with a degree of deacetylation ≥ 75.0% was used for the fabrication of AgCNCs. In the initial step, silver nanoparticles were biologically synthesized using an indigenously available fungal strain that have been previously reported to synthesize silver nanoparticles^23^. The biological method for the biosynthesis of metallic nanoparticles was preferred because of its nonhazardous and ecofriendly nature over other chemical or physical approaches. In the subsequent step, the biosynthesized silver nanoparticles were amalgamated with low molecular weight chitosan using microwave irradiation technique. Both the biosynthesized silver nanoparticles (AgNPs) and the fabricated metallic nanocomposite system (AgCNCs) were characterized in detail. Their antimicrobial potential and cytotoxicity level on mammalian cell lines were also studied. Crucially in this study, chitosan played a dual role, chitosan not only prevented the agglomeration of the nanoparticles and improved the biocompatibility but also the coating of the silver nanoparticles with this biopolymer improved the antibacterial potential of the silver nanoparticles as well.

### Biological synthesis of silver nanoparticles (AgNPs)

Silver nanoparticles could be synthesized using different physical or chemical reduction procedures and the majority of these procedures consume a large amount of energy and the nanoparticles formed during the chemical synthesis easily gets agglomerated after their formation. These large particle size formed, due to the agglomeration, could not be further classified as nanoparticles because they fall outside the nano-range. On the other hand, synthesis of silver nanoparticles using biological means is commonly considered an ecofriendly approach. Using various fungal species in comparison to conventional methods is much more preferable for this purpose and this is because excessive fungal biomass could be produced at a laboratory scale level. Customarily, during the chemical synthesis of nanoparticles, several different types of chemical agents such as silver salts (AgNO_3_), reducing agents (ethylene glycol), surfactants and stabilizers (polyvinylpyrrplidone) are exclusively required and usually are difficult to separate from the constructed nanomaterials. While in case of a biological entities, the organisms themselves are capable of generating such types of stabilizing elements (proteins, carbohydrates, amino acids etc.) in order to stabilize and regulate the rate of agglomeration of the metallic nanoparticles formed^24^. Therefore, in the current study a fungal species was selected for the extracellular biosynthesis of silver nanoparticles. Fungal biomass was used to produce silver nanoparticles under stress conditions. The preliminary confirmation of the biosynthesized silver nanoparticles was conducted using nitrate reduction test. Initially, four different strains of *Aspergillus* species were screened for the synthesis of silver nanoparticles based on the nitrate reduction intensity of each sample. Results showed that *A. fumigatus* KIBGE-IB33 was capable of reducing more silver nitrate as compared to other three species as also described earlier^23^. Silver nitrate (AgNO_3_) solution turned dark brown in color after 03 days of exposure with the fungal filtrate used whereas, the color of the positive and the negative controls remained unchanged. Therefore, *A. fumigatus* KIBGE-IB33 was selected for the synthesis of silver nanoparticles for further experimentation. The cell free filtrate of the fungal isolate that was used for the biogenic synthesis of silver nanoparticles, was also analyzed for the presence of other metabolites including carbohydrate and protein contents. The results revealed that almost insignificant concentration level of protein (3.0 µg ml^−1^) was produced in the cell free filtrate as compared to the carbohydrate content, which was approximately 74.7 mg ml^−1^. Generally, many different types of biomolecules are involved in the biosynthesis of metallic nanoparticles and among them one enzyme, known as NADPH-dependent nitrate reductase, is recognized to play a significant role as a reducing agent^25^. Therefore, it is suggested that *A. fumigatus* KIBGE-IB33 may also have metabolically synthesized this class of enzyme in very low concentrations which may have facilitated the reduction of AgNO_3_ to elemental silver and then to silver nanoparticles. This phenomenon was also confirmed in the current study by observing the change in the color of the reaction mixture from colorless to dark brown after 03 days of exposure with the fungal filtrate. Thus signaling change in the optical properties of silver nitrate solution used. Along with this, the high concentration level of total carbohydrate content produced during this process might similarly have provided additional stability to the biosynthesized nanoparticles. Hence, it is suggested that the other biomolecules formed by the selected fungal strain during this biogenic synthesis, might have also acted as capping agents that resulted in clearly dispersed nanoparticles and therefore, no agglomeration was observed. Several different fermentation factors, type of fungal strain used for production of fungal biomass and the components of the fermentation medium plays a major role in generating sufficient amounts of AgNPs. These factors are known to effect the characteristic properties of the silver nanoparticles at nanoscale range^25^. Some exopolysaccharides, produced by fungal strains, have been reported to be responsible for the reduction of AgNO_3_ to silver ions and then to silver nanoparticles. Previously, some other studies have also reported the role of bacterial, fungal and plant exopolysaccharides in the reduction and stabilization of nanoparticles^26–28^.

### Fabrication of metallic-polymer nanocomposite system (AgCNCs)

**Figure 2:**
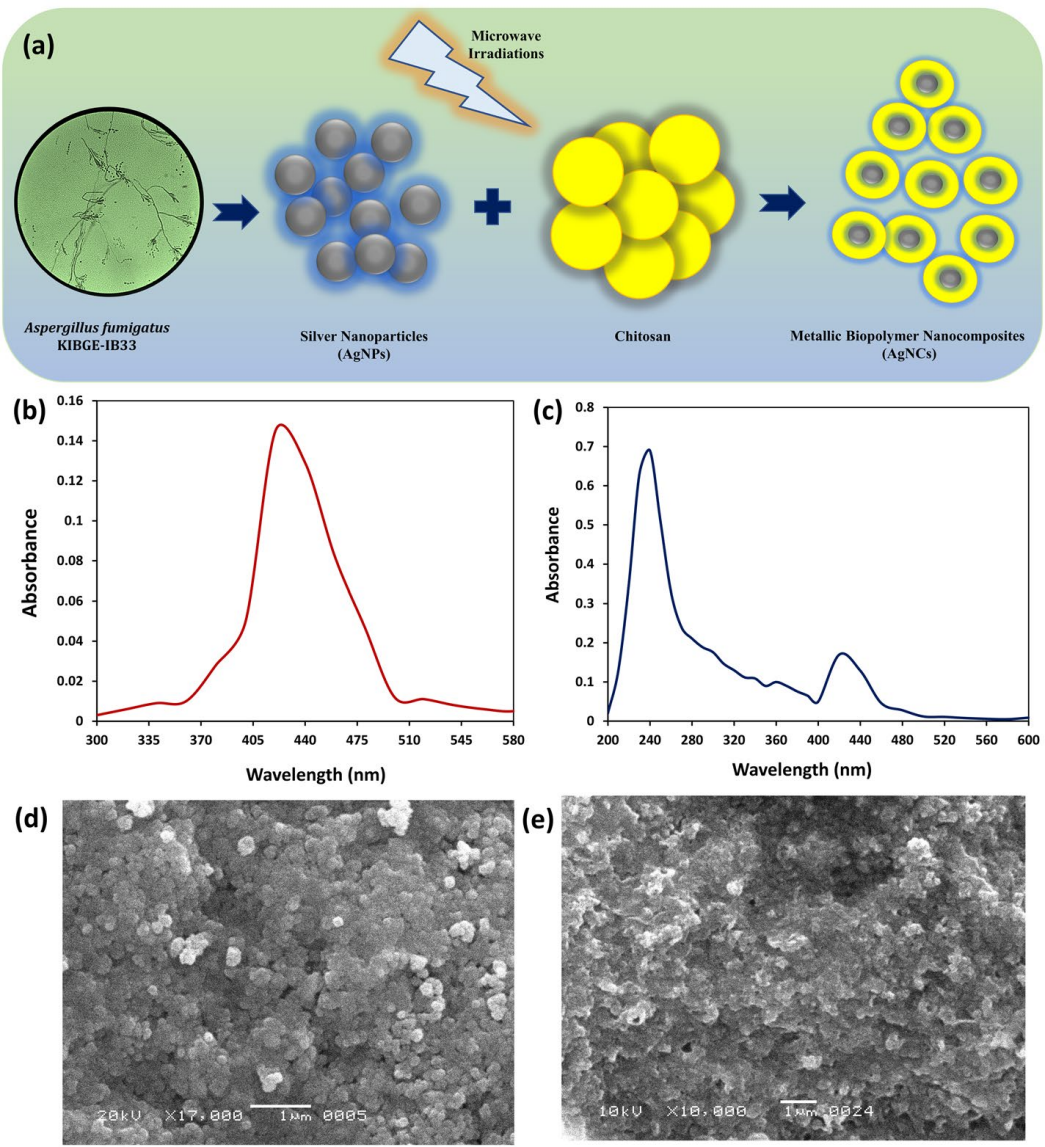
Fabrication and initial characterization of nanomaterials. (**a**) Schematic illustration of biosynthesis of silver nanoparticles (AgNPs) and fabrication of nanocomposite system (AgCNCs). (**b**,**c**) UV–visible spectra of AgNPs and AgCNCs, respectively. (**d**,**e**) surface topological analysis of AgNPs and AgCNCs, respectively using scanning electron microscope.

The pre-synthesized silver nanoparticles were used as the starter nanomaterial for the fabrication of polymer-based nanocomposite system by using a bioactive polycationic biopolymer under the influence of microwave irradiation. In the current study, low molecular weight chitosan, with a degree of deacetylation level of ≥ 75.0%, was used for the formation of metallic polymer based nanocomposites because the size of the nanocomposite system would have exceed the nanoscale range if a higher molecular weight biopolymer was used. Chitosan is a linear aminopolysacchride derived from chitin. This bioactive material has numerous applications in various fields like food packaging industry, medicines, tissue engineering and drug delivery system^29,30^. In the present study, chitosan was conjugated with the synthesized biogenic silver nanoparticles using an ex-situ polymerization approach, which is a suitable methodology for industrial level (Fig. 2a). In this one-step fabrication approach, an effective microwave heating system was used for the development of monodispersed and perfectly amalgamated metallic nanocomposites. As compared to conventional heating process, microwave irradiation is frequently used for the formation of small, uniform and evenly dispersed nanocomposites^31^. This type of fabrication procedure has become a noteworthy approach not just, because it stabilizes the metal element in a nanocomposite system but it effectively reduces the rate of agglomeration of nanomaterials. The presence of hydroxyl (OH) and amino (NH_2_) groups on chitosan which acts as functional groups binds covalently with the metallic component of the metal ions to form nanocomposites^32,33^. This outer coating of chitosan gives a characteristic feature to the developed nanocomposite system by modifying the surface morphological structure and the overall charge present on the silver nanoparticles without altering its original physicochemical characteristics.

### Characterization of biosynthesized silver nanoparticles and fabricated silver–chitosan nanocomposites

The AgNPs and AgCNCs were characterized using diverse analytical spectroscopic techniques to determine the particle size and its size distribution, surface area, shape, and crystallinity index.

**Figure 3:**
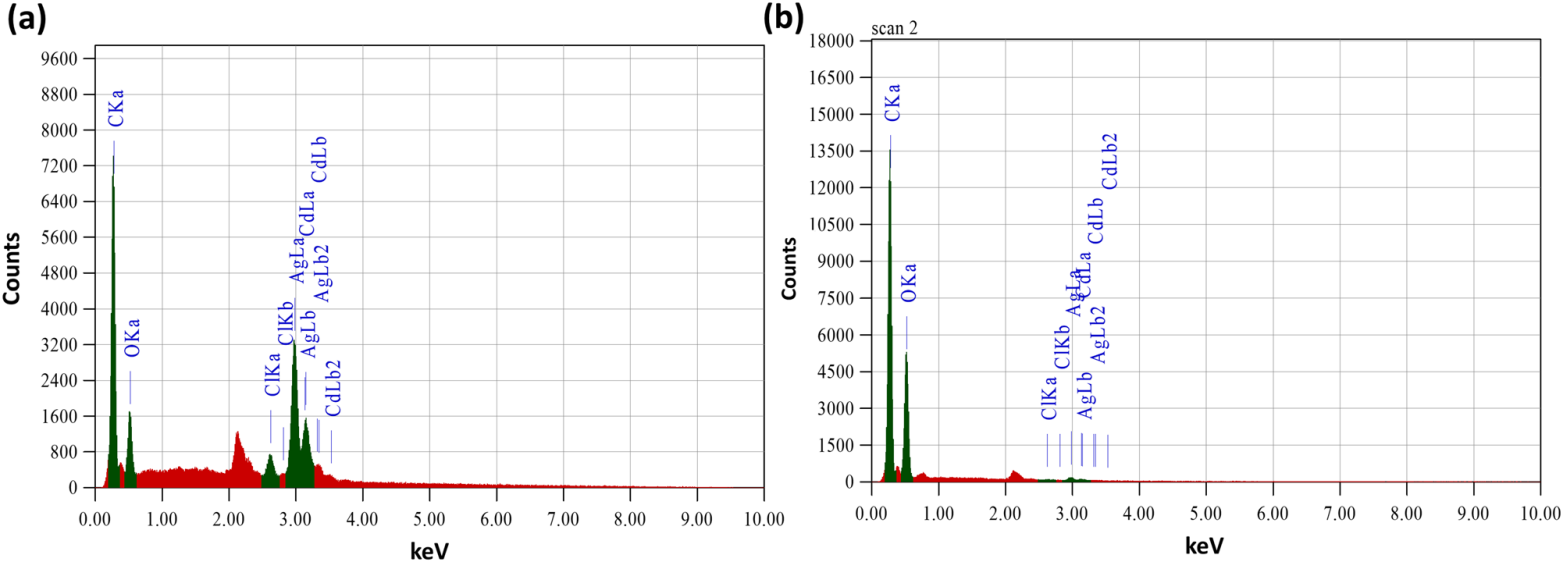
Elemental analysis of nanomaterials using energy dispersive x-ray technique. (**a**) silver nanoparticles (AgNPs) and (**b**) nanocomposite system (AgCNCs).

For the preliminary confirmation of the synthesized AgNPs and the fabricated AgCNCs, UV–visible spectroscopy was used. The maximum absorbance at 420 nm clearly confirmed the presence of silver nanoparticles (Fig. 2b) however, in case of nanocomposites two different sharp peaks were recorded at 420 nm and at 240 nm which confirmed the successful development of nanocomposite system with the coating of chitosan molecules on the silver nanoparticles (Fig. 2c). The absorption peaks at 420 nm and 240 nm are characteristic surface plasmon resonance (SPR) for AgNPs and chitosan, respectively^23,34,35^. While in another study, Biao et al. has reported an absorption peak for chitosan at 280 nm^36^. Surface topological studies of both the entities was completed using SEM. The electronic micrographs revealed the uniform, smooth and somewhat spherical shape of the AgNPs that were less than 100 nm in size (Fig. 2d). While in case of nanocomposite system, the surface of AgCNCs was rough with uneven surface topology (Fig. 2e) and this was after the layering of chitosan on the surface of silver nanoparticles that also resulted in the increase in the average particle size of AgCNCs (< 1000 nm). These initial results indicated that the surface of the nanoparticles was successfully capped by the polymeric structure of chitosan. For the confirmation of fundamental elements present in the synthesized AgNPs and AgCNCs, energy dispersive X-ray spectroscopic analysis was performed. The results showed the presence of silver, carbon and oxygen in synthesized nanoparticles and nanocomposite system with the energy levels at 0.265 keV, 0.510 keV, 2.917 keV, (Fig. 3a) and 0.277 keV, 0.525 keV, 2.983 keV, for both nano entities respectively (Fig. 3b). This data confirmed the presence of elemental silver in AgNPs and AgCNCs. Table 1 represents the chemical composition in terms of mass percentage of diverse elements that are convoluted in the foundation of both the nanostructured materials. It was evident from the data that the mass percent of elemental silver was high in AgNPs (51.6%) as compared to AgCNCs (2.85%) thus confirming the successful fabrication step. This was because AgNPs were pure silver nanoparticles and therefore, exhibited higher mass percent whereas, AgCNCs were a combination of two elements where small sum of silver nanoparticles were effectively capped with excessive mass percent of chitosan in the form of elemental carbon (56.7%).

**Table 1:** Quantitative elemental analysis of silver nanoparticles and silver nanocomposites.

Elements	Mass percent (%)	
	Silver nanoparticles	Silver nanocomposites
Carbon	25.8	56.7
Oxygen	13.8	39.5
Chlorine	1.90	0.08
Sillver	51.6	2.85
Cadmium	6.90	0.87
Nickle	ND	ND
Hydrogen	ND	ND
Total	100	100

*ND* not detected.

**Figure 4:**
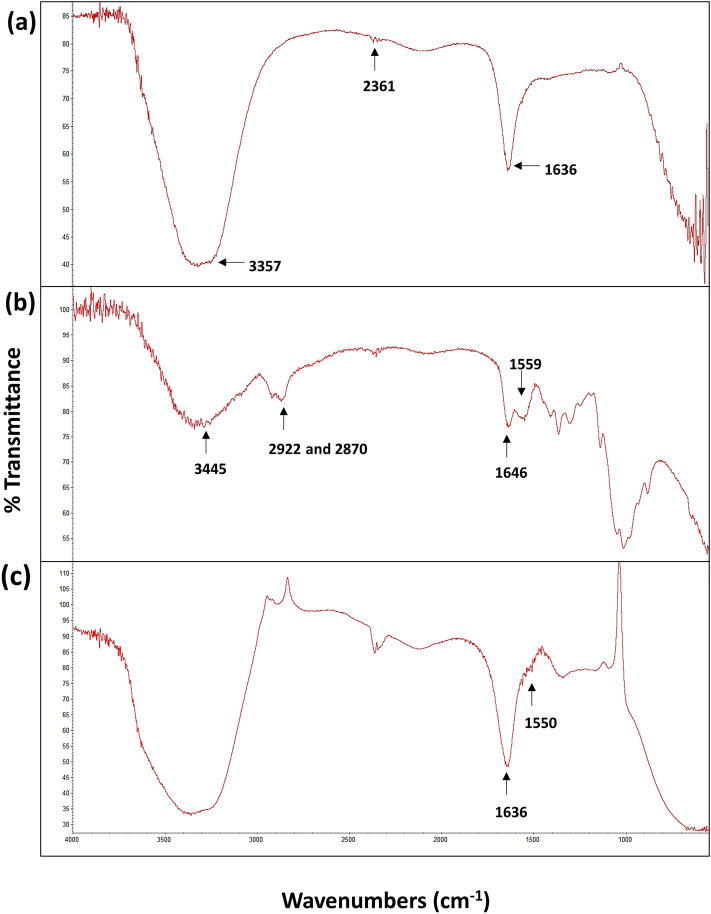
Determination of functional groups using fourier transform infrared spectroscope. (**a**) spectral analysis of silver nanoparticles (AgNPs), (**b**) spectral analysis of chitosan and (**c**) spectral analysis of silver nanocomposite system (AgCNCs).

After elemental analysis, attenuated total reflection (ATR-FTIR) spectroscopy was used to detect the functional groups present in the stabilized and functionalized nanomaterial and the nanocomposite system. The frequencies of the chemical bonds and the group of different atoms that resulted in the vibration are signifying some particular chemical bonds for both the nano entities and chitosan alone. Results revealed that the infrared spectroscopic analysis of AgNPs indicated an O–H stretching at wavenumber 3357 cm^−1^ while, N–H bending was observed at 1636 cm^−1^ (Fig. 4a). The other functional groups usually present at 1534 cm^−1^, represented the stretch vibration of C=C for alkenes or C=O stretches for amides^37,38^. However, the percent transmittance peaks of chitosan at 1060 cm^−1^ and 1646 cm^−1^ indicated the C=O carbonyl stretching and N–H bending of primary amines, respectively. Moreover, peaks at 2870 cm^−1^ and 2922 cm^−1^ are allocated to stretching vibrations of the C–H of methyl and methylene groups and at 3445 cm^−1^ showed the stretching vibration for N–H and O–H groups of chitosan (Fig. 4b) and these signals are also reported previously^36,38–40^. Notably, AgNPs exhibited two significant peaks at 1636 cm^−1^ and at 1550 cm^−1^. Though at 1550 cm^−1^, the height of the peak was reduced and this might be due to the interaction of nitrogen bonding of primary amine groups and amide groups of chitosan to the silver nanoparticles (Fig. 4c). These interactions correspondingly reduced the carbonyl stretching (C=O) and the deformation vibration of N–H groups.

**Figure 5:**
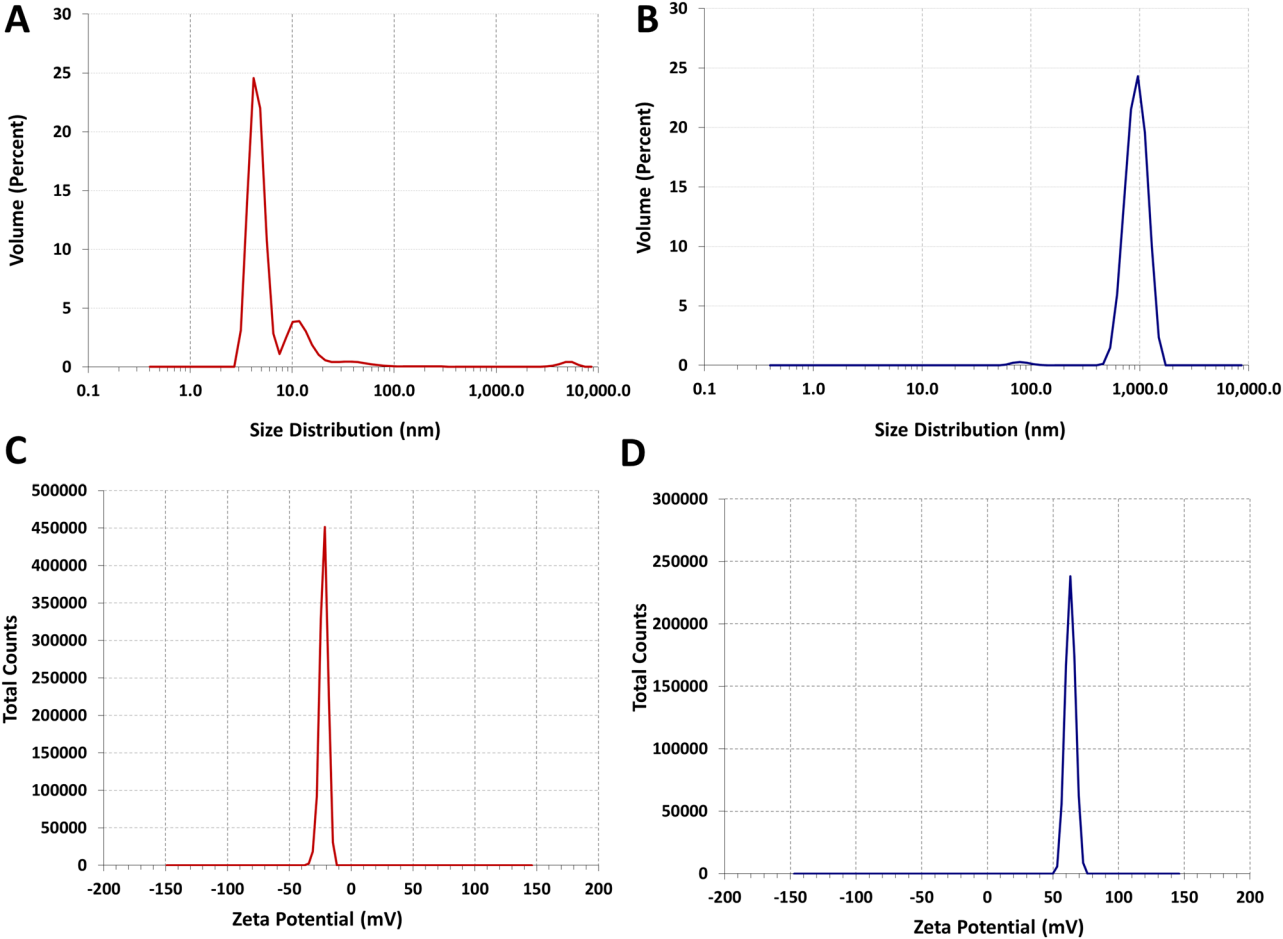
Average particle size distribution and surface charge values of the developed nanomaterials. (**a**,**b**) particle size distribution of silver nanoparticles (AgNPs) and silver nanocomposite system (AgCNCs) estimated using dynamic light scattering, respectively. (**c**) and (**d**) zeta potential values of silver nanoparticles (AgNPs) and silver nanocomposite system (AgCNCs) calculated using dynamic light scattering, respectively.

Initially, SEM was used to observe the surface morphology however, this techniques also provided a rough estimation of the particle size for both the nano entities. However, for the accurate determination of the average hydrodynamic diameter of the biogenic nanomaterial and the fabricated nanocomposite system, a more sensitive technique of DLS was used. The results of the Brownian motion of the distributed nanoparticles and the nanocomposites obtained from DLS spectroscopy showed that the overall average particle size of the AgNPs was in the range between 01 and 50 nm in size (Fig. 5a). The most prominent average particle size of the silver nanoparticle recorded was 05 nm (77.0%) whereas, 13 nm and 48 nm particle sizes were also recorded in the range of 19.0% and 4.0%, respectively with a polydispersity index (PDI) of 0.4. The current data of zeta average also suggests that the biogenic silver nanoparticles are polydisperse in nature. This PDI value and very small nano size (05 nm) of AgNPs biosynthesized in bulk, emphasize their plausible applications as anti-angiogenic, anticancer and as an antiviral agent^41–43^. The molar concentration of the biosynthesized AgNPs (05 nm) was also calculated using the Eqs. (1) and (2) and it was estimated to be approximately 1.825 mM n solution, when 3.5 mM of AgNO_3_ was used. On the other hand, about 99.0% of the metallic polymer-based nanocomposites were in the average size of 941 nm, which represents the successful coating of the silver nanoparticles with the selected biopolymer (Fig. 5b). The PDI value for this nanocomposite system was recorded as 0.573. The particle size of AgCNCs might vary depending upon the molecular weight and the degree of deacetylation of the chitosan used for the capping of nanomaterials and may also enhance the physicochemical properties of the nanoparticles^44,45^. The overall surface charge of the nanomaterials was also determined by calculating its zeta potential value using DLS. The zeta potential of the AgNPs was − 22.1 mV and for AgCNCs was + 63.2 mV (Fig. 5c,d). The negative charge of the silver nanoparticles demonstrates their well-dispersed behavior in the medium and reflects strong repulsion between the silver nanoparticles thus, confirming their stability in the liquid medium with high surface energies. This positive nature of the nanocomposite system was due to presence of the protonized amino group (–NH^3+^) of the chitosan molecule which also further confirmed the successful protective coating of the chitosan on the surface of the AgNPs. This capping of biopolymer not only assisted in the reduction of the agglomeration of AgNPs but also stabilized the even dispersion of the particles in Ag-chitosan nanocomposite system.

### Long-term efficiency and stabilization of nanocomposite system

**Figure 6:**
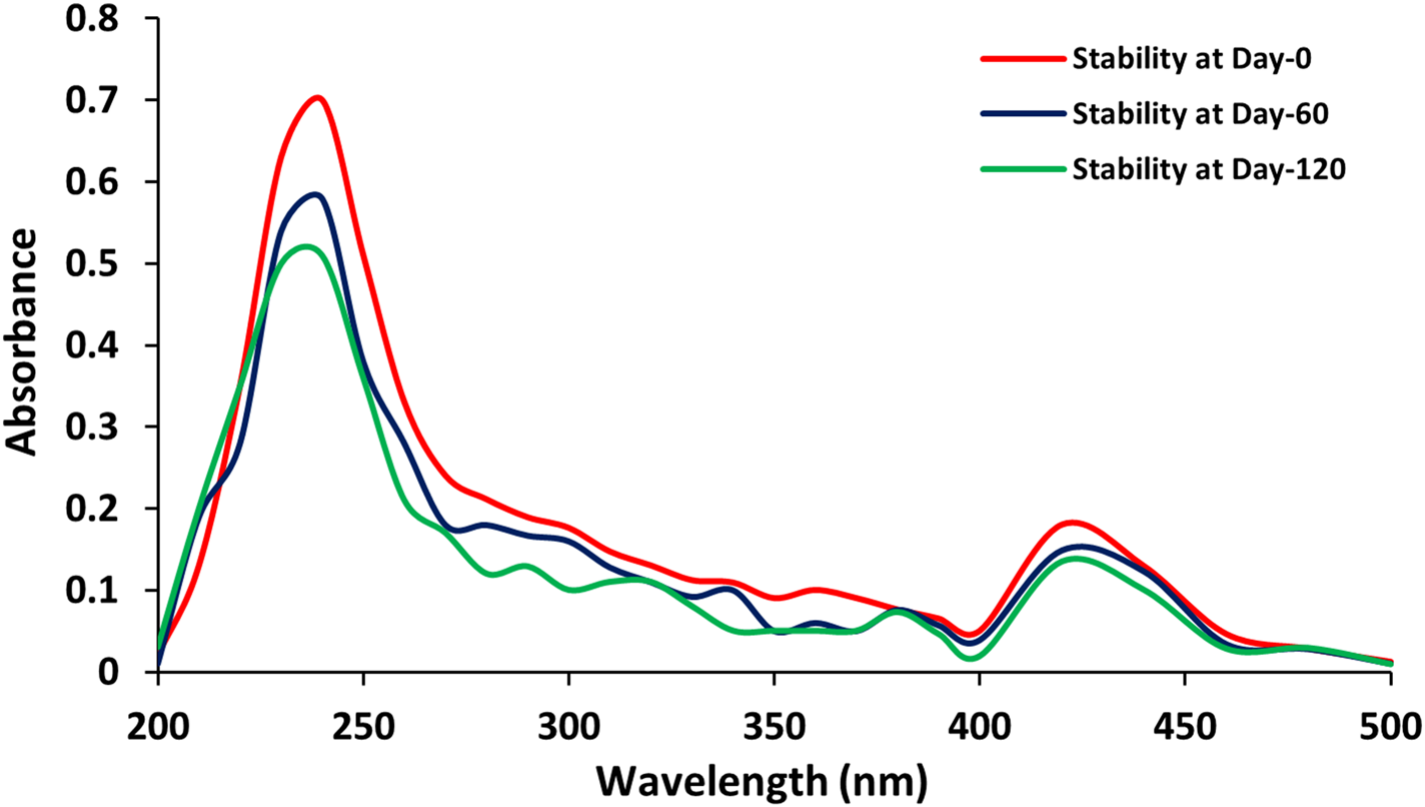
Long term storage stability of the developed nanocomposite system (AgCNCs).

UV–Visible absorption spectrum was monitored to study the long-term stability of both silver nanoparticles and the Ag-chitosan nanocomposites for different time intervals. The recorded spectral peaks at 240 nm and 420 nm represented the absorption for silver nanoparticles and chitosan, respectively (Fig. 6). The results displayed no prominent change or alteration in the absorption spectra for both the peaks even after 120 days when the nanocomposites were stored at 4 °C. Moreover, the stability of the silver nanoparticles was also determined using the same method and they exhibited stability up to only 90 days (data not shown) as compared to nanocomposite system developed. Usually in chemical approach, thermodynamically unstable nanomaterials may form aggregates due to Oswald ripening therefore, additional stabilizing agents are required to stabilize these nanomaterials. However, in case of biological synthesis, the biological system itself produces some types of exopolysaccharides or proteins that not only reduce the chemical compounds but also simultaneously provides stability to the biosynthesized nanoparticles. The most suitable way to stabilize bare silver nanoparticles is to use electrostatic stabilization method. The bioactive and biocompatible molecules such as biopolymers are mostly preferred to stabilize metallic nanoparticles. The low molecular weight polycationic linear aminopolysaccharide (chitosan), which was used to develop the silver polymer-based nanocomposite system, must have provided additional stability to it and also prevented aggregation^45^. The Ag-chitosan nanocomposites developed in the present study are more stable in nature and can be stored in suspension form for up to 120 days without incorporation of any other additional stabilizer. In another study, the stability of silver/titanium dioxide chitosan nanocomposites was reported for up to 60 days only^46^.

### Antimicrobial potential of nanomaterial and nanocomposite system

After physicochemical characterization, antimicrobial potential of silver nanoparticles and fabricated nanocomposite system was studied against various multidrug resistant microorganisms (MDROs). Table 2 demonstrated the antimicrobial potential of AgNPs and AgCNCs along with the calculated MIC and FIC values of nanocomposite system only against some prominent pathogenic indicator strains. Results indicated that AgCNCs exhibited strong antibacterial activity against both gram-negative bacteria and gram-positive bacteria as compared to chitosan and AgNPs alone. However, unfortunately all these three entities (AgNPs, chitosan and AgCNCs) did not displayed any antifungal action. The bare AgNPs biosynthesized in the present study alone themselves also exhibited notable antibacterial effect as a biocidal agent and this might be due to the very small size (05 nm) of the AgNPs and their increased surface area. This distinct nanoscale sized silver nanoparticles might have been easily up taken by the microbial cells and therefore had a higher dissolution effect on the selected microorganisms. Similarly, the nanocomposite system displayed a much profound effect on all the selected microorganisms as compared to the bare AgNPs and this is because of the conjugated biopolymer with the AgNPs. Chitosan has active functional groups (amino and hydroxyl groups) in its structure which are known for their biocidal activities. The possible mechanism here may involve the interference of the poly-cationic nature of the chitosan with the negatively charged residues present on the cell surface of the microorganisms^47^. However, some other factors could have also been involved in antibacterial activity and these includes the molar mass or the size, density of the positive charge present, hydrophilicity/hydrophobicity and also the ionic strength of the chitosan used for the fabrication of nanocomposite system^48,49^.

**Table 2:** Antimicrobial potential of biosynthesized silver nanoparticles and silver nanocomposites along with inhibitory concentrations of silver nanocomposites.

Microorganisms used as indicator strains	Antibacterial activity (zone of inhibition in mm)		
	Silver nanoparticles	Chitosan	Silver nanocomposites
*Enterococcus faecalis* ATCC 29212^a^	11 ± 0.22	13 ± 0.26	20 ± 0.4
*Salmonella typhimurium* ATCC 3632^b^	11 ± 0.22	5 ± 0.1	18 ± 0.36
*Pseudomonas aeruginosa* ATCC27853^b^	13 ± 0.26	2 ± 0.04	18 ± 0.36
*Listeria monocytogenes* ATCC 7644^c^	12 ± 0.24	5 ± 0.1	15 ± 0.3
*Escherichia coli* ATCC 8739^c^	11 ± 0.22	5 ± 0.1	13 ± 0.26
Methicillin resistant *Staphylococcus aureus* KIBGE-IB23^b^	9 ± 0.18	ND	10 ± 0.2
*Bacillus cereus* ATCC 11778^b^	9 ± 0.18	ND	8 ± 0.16
*Aspergillus flavus* KIBGE-IB34^d^	ND	ND	ND
*Aspergillus terreus* KIBGE-IB35^d^	ND	ND	ND
*Aspergillus niger* KIBGE-IB36^d^	ND	ND	ND
*Aspergillus fumigatus* KIBGE-IB33^d^	ND	ND	ND

Microorganisms		Minimum Inhibitory Concentrations (µg ml^−1^)	
**Minimum inhibitory concentration of silver nanocomposites**			
*Enterococcus faecalis* ATCC 29212^a^		1.56 ± 0.03	
*Salmonella typhimurium* ATCC 3632^b^		3.12 ± 0.06	
*Listeria monocytogenes* ATCC 7644^c^		7.8 ± 0.15	
*Pseudomonas aeruginosa* ATCC 27853^b^		12.5 ± 0.25	

Microorganisms	Fractional inhibitory concentration index (FICI)	Combinatorial effect	
**Fractional inhibitory concentration index of silver nanocomposites**			
*Enterococcus faecalis* ATCC 29212^a^	0.02	Synergistic effect	
*Salmonella typhimurium* ATCC 3632^b^	0.40	Synergistic effect	
*Listeria monocytogenes* ATCC 7644^c^	0.03	Synergistic effect	
*Pseudomonas aeruginosa* ATCC27853^b^	0.06	Synergistic effect	

ND: Zone of inhibition not detected.

^a^Microorganism was cultivated at 37 °C for 04 h (log-phase) in nutrient broth.

^b^Microorganism was cultivated at 37 °C for 03 h (log-phase) in nutrient broth.

^c^Microorganism was cultivated at 37 °C for 02 h (log-phase) in nutrient broth.

^d^Microorganism was cultivated at 30 °C for 72 h in potato dextrose broth.

For quantitative analysis, minimal inhibitory concentration (MIC) of nanocomposite system was also calculated. In the current study, the lowest MIC for AgCNCs was noticed against *Enterococcus faecalis* ATCC 29212 with an MIC of 1.56 µg ml^−1^ (Table 2). While, MIC values against *Salmonella typhimurium* ATCC 3632, *Listeria monocytogenes* ATCC 7644 and *Pseudomonas aeruginosa* ATCC 27853 was almost 3.125 µg ml^−1^, 7.8 µg ml^−1^ and 12.5 µg ml^−1^, respectively. Formerly, chitosan fabricated with silver nanoparticles have displayed 2.48 µg ml^−1^ MIC against *Escherichia coli*^36^. Similarly, the MIC of 4.96 µg ml^−1^ of silver nanocomposites was reported against *Staphylococcus aureus*^48^.

The current data acquired from the MIC was also utilized to calculate the fractional inhibitory concentration (FIC) in order to conclude if the biosynthesized silver nanoparticles and the polymer both have any combined synergistic effect as a fabricated nanocomposite system or not. Previously, it has been reported in the literature that is the FIC index values are ≥ 2.0 then it specifies an antagonistic effect, while the values between 0.5 and 2.0 designate an additive effect and if the FIC values are ≤ 0.5 than it will indicate a synergistic behavior between the two selected entities^50–52^. Current data revealed that the FICI of silver nanocomposite system (AgCNCs) was less than 0.5 against all the four tested indicator strains confirming the synergistic behavior of both the components of nanocomposites (Table 2). These results suggested that the fabricated metallic polymer-based nanocomposites have a potential to be used as an alternative approach for the treatment of MDROs with enhanced antibacterial activity when used in combination. Similarly, antipathogenic activity of different AgNPs using two *Pseudomonas* pathogen strains has also been previously reported^53^.

### In vitro analysis of silver nanoparticles and nanocomposite system

**Figure 7:**
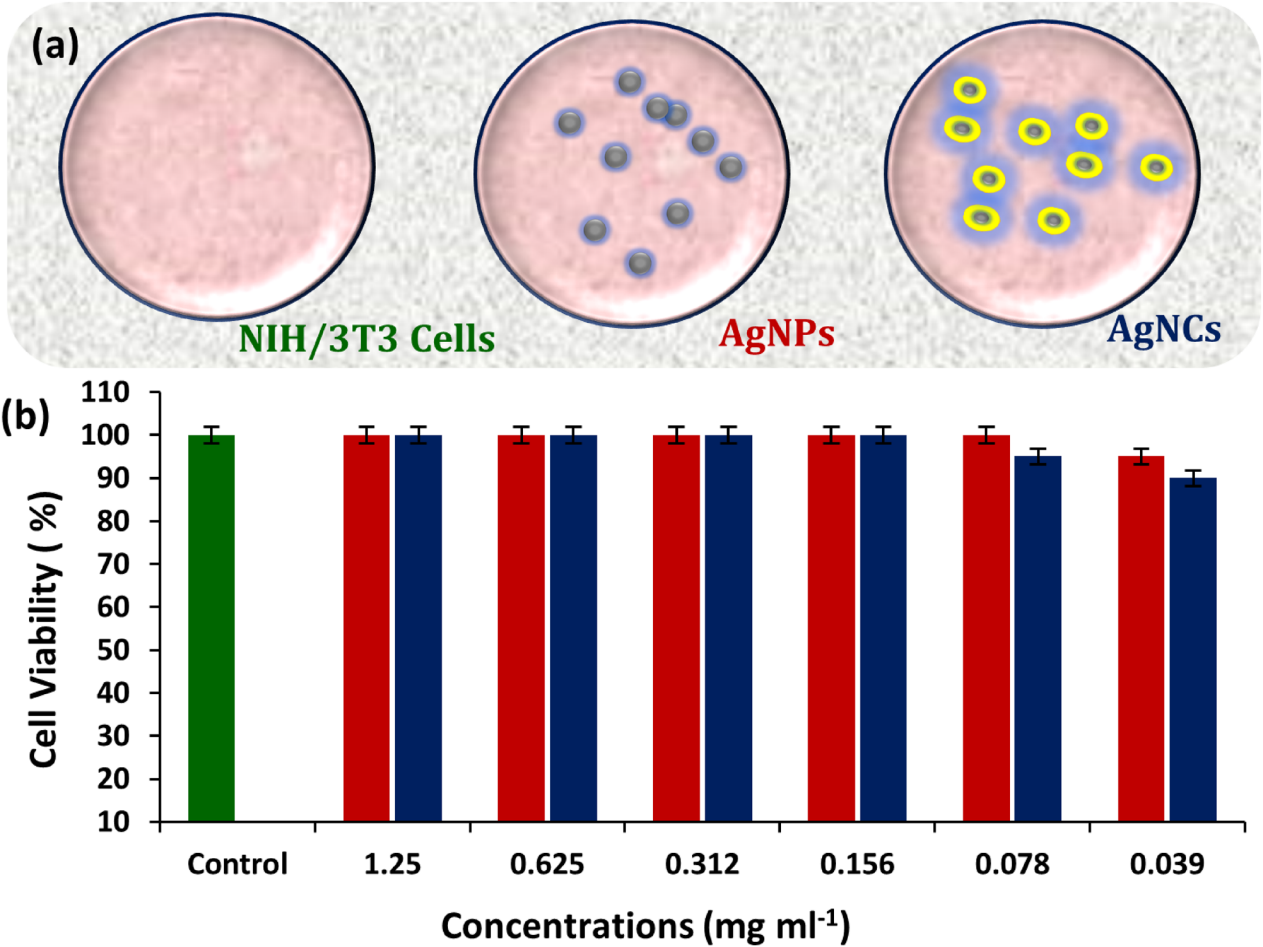
Cell cytotoxicity analysis on the viability of NIH/3T3 cells using MTS assay. (**a**) schematic presentation of cell viability. (**b**) effect of different concentrations of the developed silver nanoparticles (AgNPs) and the silver nanocomposite system (AgCNCs).

The cytotoxic effect of the biosynthesized and the fabricated nanomaterials was studied. Cell cytotoxicity assay was performed to determine the cytotoxic effect of the biosynthesized AgNPs and AgCNCs on NIH/3T3 mouse fibroblast cells. In this assay, mammalian cell lines were treated with various concentrations of nanoparticles and nanocomposites. The results revealed that more than 90.0% of NIH/3T3 mouse fibroblast cells could survived even in the presence of high concentrations of both the compounds thus confirming the nontoxic nature of the AgNPs and AgCNCs (Fig. 7a,b). These murine cell lines were able to survive all the tested concentrations. Moreover, these nontoxic concentrations are much higher than the minimal inhibitory concentrations required for the tested compounds to exterminate the pathogenic multidrug resistant organisms. Therefore, it can be concluded that AgNPs and AgCNCs could be utilized in future in the form of nanomedicine after some quantifiable trials, as they are nontoxic and antibacterial in nature even in low doses.

## Conclusions

In the current study, green synthesis approach was used for the formation of silver nanoparticles which were further used for the fabrication of silver–chitosan based nanocomposite system. To best of our knowledge, this study first time reports the biosynthesis of very small size of the silver nanoparticles (05 nm) thus exhibiting the novelty of the approach adapted. The biosynthetic methodology designed in this study resulted in the formation of nanoparticles that not only exhibited the antibacterial potential alone but also expressed improved antibacterial spectrum in synergism with a biopolymer. This study resulted in the production of some stabilizing exopolysaccharides that not only provided long-term stability to the silver nanoparticles but also successfully prevented their agglomeration. Further successful coating of these nanoparticles with chitosan resulted in their extended stability in suspension form and suggests that the particle charge on the developed nanocomposite system could be used for interaction with different type of chemical compounds. The antibacterial potential and the synergistic behavior of both the nano entities against some multidrug resistant microorganisms with almost no cytotoxic effect, seamlessly accentuates their potential application in the field of medicine as a nanocarrier system.

## Methods

### Materials and chemicals

Extra pure silver nitrate, 99.0% (Cat No. PL00490100, Scharlau, Spain), acetic acid puriss, 99.8% (Cat No. 27225, Sigma-Aldrich) and chitosan from shrimp shells ≥ 75.0% degree of deacetylation (Cat No. C3646-1009, Sigma-Aldrich) were used throughout this research work. Medium components were from Oxoid, UK. Dulbecco′s Modified Eagle′s Medium (DMEM, Cat No. 11965-092, Gibco, Life Technologies), Fetal Bovine Serum (FBS, Cat No. 10500-064, Gibco, Life Technologies), Penicillin/Streptomycin (Pen : Strep, 10,000 Units ml^−1^:10,000 µg ml^−1^, Cat No. 15140-122, Gibco, Life Technologies) and Cell Titer 96 Aqueous One Solution Cell Proliferation Assay (Cat No: G3580, Promega , USA) were also used in the study. All the chemicals were of analytical grade. Double deionized water was used for the preparation of regents otherwise specified.

### Screening of nitrate reducing microorganisms

Four indigenously isolated *Aspergillus* species were screened for their ability to reduce nitrate salt^54^. All the strains were cultivated in potato dextrose broth (PDB) that consist of (gl^−1^): potato starch, 0.3 and dextrose, 20.0 (pH: 5.6 ± 0.2). All the cultures were incubated at 30 °C for 72.0 h and were processed for the screening of nitrate reduction. After 72.0 h fungal biomass was harvested using Whatman filter paper No. 1 and the cell free supernatant (100 µl) was mixed with silver nitrate (1.0 mM) and the reaction was carried out in dark at 30 °C for up to 3.0 days. The change in color from white to brown was visualized for the reduction of silver nitrate to silver.

### Production of fungal biomass for the biosynthesis of silver nanoparticles

For the biosynthesis of silver nanoparticles, previously described method with slight modifications was used^23^. Fungal strain that displayed maximum reduction of silver nitrate was selected for further studies. For the production of fungal biomass, *Aspergillus fumigatus* KIBGE-IB33 was sub-cultured in PDB (1000 ml) at 30 °C for 05 days. Fungal biomass was separated using Whatman filter paper No. 1 and inoculated in sterilized double deionized water (2500 ml). This suspension was kept at 30 °C for 03 days under static condition. After three days, fungal biomass was separated by ultracentrifugation (40,000×*g*) at 4 °C for 15 min under sterilized condition. This cell free extract was further used for the biosynthesis of silver nanoparticles. For this purpose, silver nitrate (3.5 mM) was incorporated in the cell free filtrate and was again kept in dark with orbital shaking (100 rpm) at 25 °C for 03 days. After 3 days, the suspension containing biosynthesized silver nanoparticles was subjected to characterization of the developed silver nanoparticles and for the subsequent fabrication of silver nanocomposites.

Molar concentration of the biosynthesized silver nanoparticles was calculated by a method described earlier^55^. For this purpose, first the concentration of the silver nanoparticles was calculated by the following formula:

1 $$N = \frac{{\pi \rho D^{3} }}{{6{\rm M}}}{ }{\rm N}_{\rm A}$$

where *N* is the number of atoms per nanoparticles; the value of π is 3.14; the value of *ρ* is the density of face centered cubic (fcc) silver (10.5 g cm^−3^); *D* is the average diameter of nanoparticles (~ 05 nm = 5 × 10^−7^ cm); *M* is atomic mass of silver (107.868 g); *N*_*A*_ is the Avogadro’s number [atoms per mole (6.023 × 10^23^)].

While, for the calculation of molar concentration of the biosynthesized silver nanoparticles in solution^56^, the following formula was used:

2 $${\text{C}} = \frac{{{\text{N}}_{{\text{T}}} }}{{NVN_{A} }}$$

where *C* is the molar concentration of nanoparticles in solution; *NT* is the total number of silver atoms added as AgNO_3_ = 1.0 M; *N* is the number of atoms per nanoparticle (calculated from Eq. 1); *V* is the volume of the reaction solution in liter; *NA* is the Avogadro’s number (6.023 × 10^23^).

### Determination of total protein and carbohydrate content of cell free extract

The cell free extract containing biosynthesized silver nanoparticles was examined for total protein and carbohydrate contents. Lowry’s Assay was performed to determine the total protein concentration of the extract and Anthrone’s method was used for the determination of carbohydrate content^57,58^.

### Fabrication of silver–chitosan nanocomposites

Biosynthesized silver nanoparticles were used to fabricate silver nanocomposites and the biopolymer used for this purpose was low molecular weight chitosan (≥ 75% degree of deacetylation). The method used for the fabrication of metallic-polymer based nanocomposite was adapted from a previously described protocol with some modifications^59^. Chitosan (15.0 gl^−1^) was solubilized in glacial acetic acid (1.0%). The suspension containing silver nanoparticles was mixed with this chitosan solution (1:2 *v/v*). The reaction mixture was exposed to microwave heating at 650 W for 90 s with 30-s intervals and was cooled at room temperature. This suspension containing fabricated nanocomposite system was stored at 4 °C for further characterization.

### Confirmation of silver nanoparticles and nanocomposite system

Initially for the detection and confirmation of the biosynthesized silver nanoparticles and the fabricated silver–chitosan nanocomposites, UV–Visible spectrophotometry was used. Absorption spectra for both the samples were measured using a spectrophotometer (UVS-2700, Labomed Inc., USA). The spectral range used for the detection of both samples was in the range between 200 and 700 nm.

### Surface topology of silver nanoparticles and nanocomposite system by scanning electron microscopy

The surface morphology and shape of the silver nanoparticles and the fabricated silver nanocomposites were observed through scanning electron microscope (SEM) (JSM 6389 A Jeol, Japan). The dried samples were deposited on the SEM stub which was coated with gold (Au) and was focused up to 300 Å in an auto coater (JFC-1500 Jeol, Japan). The scans were observed until a better resolution of the samples was achieved.

### Elemental analysis of silver nanoparticles and nanocomposite system by energy dispersive x-ray spectroscopy

The biosynthesized silver nanoparticles and the fabricated silver nanocomposites were subjected to energy dispersive X-ray (EDX) spectroscopy for the detection of the fundamental elements.

### Functional groups analysis of silver nanoparticles and nanocomposite system by Fourier transform infrared spectroscopy

Fourier Transform Infrared (FTIR) spectrum analysis of both biosynthesized silver nanoparticles and the fabricated silver nanocomposites were determined through FTIR (Thermo Fisher, USA/Nicolet iS5) in the scanning range from 400 to 4000 cm^−1^ using an ATR mode of operation.

### Determination of zeta potential value and size distribution of silver nanoparticles and nanocomposite system by dynamic light scattering spectroscopy

Zeta potential and particle size distribution of the biosynthesized silver nanoparticles and the fabricated silver nanocomposites were estimated using dynamic light scattering (DLS) spectroscopic analysis. All the measurements were conducted at 25 °C using a DLS system (LM10. Malvern Zetasizer, United Kingdom). The Brownian motion was studied and the distributed particle size in a given sample was calculated using Stokes–Einstein relationship^60^.

### Storage stability of the fabricated nanocomposite system

Long-term storage stability of the fabricated silver nanocomposites was analyzed by UV–visible spectrophotometer within the wavelength range from 200 to 500 nm. The fabricated silver nanocomposites were stored at 4 °C and their spectrum was determined for different time intervals up to 03 months.

### Minimal inhibitory and fractional inhibitory concentrations of silver nanoparticles and nanocomposite system

Agar well diffusion technique was carried out to determine the antimicrobial potential of the biosynthesized silver nanoparticles and the fabricated silver nanocomposites against various pathogenic bacterial and fungal strains. All the indicator bacterial species were incubated at 37 °C in nutrient broth and the log phase (10^8^ CFU ml^−1^) of each culture were streaked on nutrient agar plates. While all the fungal strains were cultivated at 30 °C on potato dextrose broth (PDB). Two-fold serial dilutions were prepared in normal saline and the fungal biomass was mixed in molten potato dextrose agar before pouring the suspension in the petri plates. The samples were added in already prepared 05 mm wells in agar medium and the petri plates were incubated under standard conditions according to the type of bacterial culture used. The data for minimum inhibitory concentration (MIC) was estimated using micro dilution methods as described earlier by Clinical and Laboratory Standards Institute^61^. Dilutions (1:1) of metal nanoparticles and the biopolymer alone were used as controls. Minimal inhibitory concentration (MIC) was recorded by measuring the zone of inhibition in millimeters. All the experiments were performed in triplicates and results were the mean of the three independent values. Fractional inhibitory concentration (FIC) was also calculated for the determination of synergistic behavior of silver nanoparticles and chitosan. For this purpose, previously calculated minimal inhibitory concentrations of the silver nanoparticles and chitosan biopolymer were used in the given equation as described earlier^50,62^ and fractional inhibitory concentrations were interpreted using the following FIC index equation:

3 $$FIC\; Index = \left[ {MIC\; A\left( {Combined} \right)/MIC\; A\left( {Alone} \right)} \right] + \left[ {MIC \;B\left( {Combined} \right)/MIC\; B\left( {Alone} \right)} \right]$$

### Cell viability assay

The cytotoxicity effect of biosynthesized silver nanoparticles and the fabricated silver nanocomposites was determined on NIH/3T3 mouse fibroblast cell lines (ATCC CRL-1658TM) maintained in DMEM with 10.0% heat inactivated FBS along with 1.0% of streptomycin-penicillin. After achieving 80.0% confluency level, 5 × 10^3^ cells were seeded in each well of the 96 well plate and incubated at 37 °C for 24 h under 5.0% carbon dioxide. After 24 h of incubation, cells were treated with different concentrations of synthesized nanoparticles and nanocomposites and incubated for further 24 h. Afterwards MTS reagent was added and after 02 h of incubation, the absorbance was recorded at 490 nm. Positive control (Triton X-100) and negative control (DMEM) were also prepared. The viability of cells was measured in terms of percentage using the following equation:

4 $$Cell \;viability \left( \% \right) = \frac{O.D. \;of\;test}{{O.D. \;of\; control}} \times 100$$

### Statistical analysis

All the experiments were performed three times in triplicates and the data is presented as the mean value of all with a ± 2.0 value for standard deviation (SD). The *P* value (< 0.05) was considered as statistically significant after determining the differences between the data sets using Student’s t-test.

## Data Availability

All data and results are available upon request.
